# Moslae Herba: Botany, Traditional Uses, Phytochemistry, and Pharmacology

**DOI:** 10.3390/molecules29081716

**Published:** 2024-04-10

**Authors:** Zhuo-Ying Duan, Yan-Ping Sun, Zhi-Bin Wang, Hai-Xue Kuang

**Affiliations:** Key Laboratory of Basic and Application Research of Beiyao, Ministry of Education, Heilongjiang University of Chinese Medicine, Harbin 150040, China; 18729999509@163.com (Z.-Y.D.); sunyanping_1@163.com (Y.-P.S.)

**Keywords:** Moslae Herba, *Mosla chinensis* Maxim, *Mosla chinensis* ‘Jiangxiangru’, phytochemistry, pharmacology

## Abstract

Moslae Herba (MH) can be used for both medicine and food and has a long history of medicine. MH has the effects of sweating and relieving the exterior, removing dampness and harmonizing, and is mainly used for colds caused by damp heat in summer. It is called “Xiayue Zhi Mahuang” in China. So far, 123 chemical compounds have been isolated and identified from MH, including flavonoids, terpenoids, phenolic acids, phenylpropanoids, and other chemical compounds. Its chemical components have a wide range of pharmacological activities, including antibacterial, antiviral, anti-inflammatory, antioxidant, analgesic sedation, antipyretic, immune regulation, insecticidal, and other effects. In addition, because of its aromatic odor and health care function, MH also has development and utilization value in food, chemical, and other fields. This paper reviewed the research progress of MH in botany, traditional uses, phytochemistry, and pharmacology and provided a possible direction for further research.

## 1. Introduction

Mosla Herba (MH) is the aboveground dry part of the genus *Mosla* plants *Mosla chinensis* Maxim (*M. chinensis* Maxim) (www.worldfloraonline.org (accessed on 6 February 2024) and its cultivated variety *Mosla chinensis* ‘Jiangxiangru’ (*M. chinensis* ‘Jiangxiangru’), mainly distributed in southern China and northern Vietnam [1]. It is used as food by Chinese people in spring and summer and is also a traditional Chinese medicine (TCM) with a long history. It tastes pungent and warm and belongs to lung and stomach meridians.

It has the function of sweating and relieving the surface, dispelling dampness and mediating, and is often used to treat summer-dampness cold, abdominal pain, vomiting, and diarrhea [2]. It is first listed in “Ming Yi Bie Lu” (A.D. 220–450) and listed as the medium grade. Xiangru san was first discovered in the Song Dynasty “Prescriptions People’s Welfare Pharmacy”, and later generations of doctors added and subtracted many new prescriptions on this foundation. Looking up ancient and modern materials, it is found that the medicinal plants of MH have undergone some changes in history. The original plants recorded before the Song Dynasty were more consistent with *Elsholtzia ciliate*, which was mainly used to treat heat stroke and cholera. In the Ming and Qing dynasties, *M. chinensis* Maxim, a new variety, rose to the mainstream position because of its remarkable efficacy and the formation of cultivation, and differentiated into the cultivated product *M. chinensis* ‘Jiangxiangru’. After that, *Elsholtzia* species were gradually eliminated. As for the later *Mosla* varieties, later generations of doctors generally believe that it has a strong sweating ability. The treatment of heat stroke and cholera patients should be based on syndrome differentiation and careful medication [3]. Since the 2005 edition of the Chinese Pharmacopoeia, China has stipulated that the original plants of MH are *M. chinensis* Maxim and *M. chinensis* ‘Jiangxiangru’ of the genus *Mosla*, which have continued to this day [4]. In recent decades, there have been many complicated studies on MH plants. This paper discusses genuine products of MH based on the 2020 edition of ‘Chinese Pharmacopoeia’. Phytochemical studies have shown that there are multiple active components in MH, which can be roughly divided into volatile components and non-volatile components. Researchers have conducted a lot of studies on MH volatile oils by gas chromatography-mass spectrometry (GC-MS) and identified more than 200 volatiles represented by thymol and carvacrol. So far, about 123 compounds have been isolated and identified from MH, mainly divided into flavonoids, terpenoids, phenolic acids, and phenylpropanoids [5,6,7,8]. Flavonoids and terpenoids are the two compounds with the highest proportions among the isolated compounds. As people pay increasing attention to the pharmacological activity of MH, researchers have found that MH has prominent antibacterial, antiviral, antioxidant, anti-inflammatory, analgesic and sedative, antipyretic, immune enhancement, insecticide, and other effects [9,10,11,12,13,14]. Some scholars also tried to explore its potential pharmacological activity through molecular docking technology [15]. Due to its aromatic smell, abundant volatile oils, and extensive biological activity, some studies have reported the potential development value of MH in the chemical industry, perfume, health care, and other fields.

**Figure 1:**
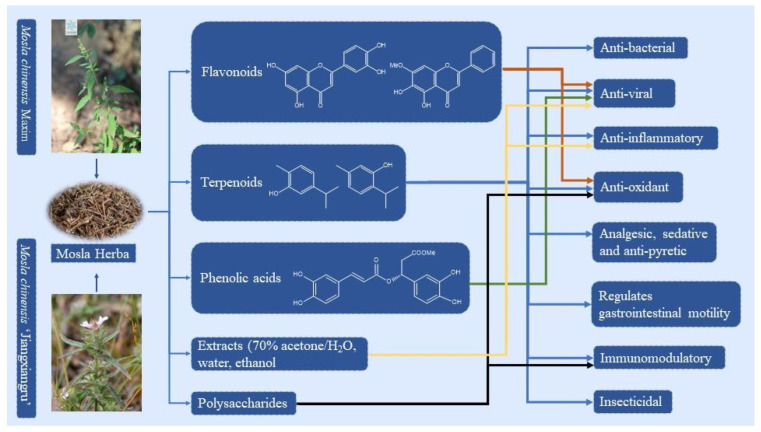
The known components and pharmacological effects of MH (the image of MCM is from the Plant Photo Bank of China).

However, *M. chinensis* Maxim and *M. chinensis* ‘Jiangxiangru’ are closely related and generally difficult to identify. To this end, some scholars suggested that megakaryocyte-associated tyrosine kinase (matK) and internal transcribed spacer 2 database (ITS2) sequences could be used as DNA barcodes to identify MH and its adulterants. The experimental results showed that there is an obvious barcoding gap between the authentic MH and its adulterants. And *M. chinensis* Maxim and *M. chinensis* ‘Jiangxiangru’ can be distinguished at the primary nucleic acid sequence level of matK and ITS2 [16]. This attempt provides a new method for the accurate identification of MH and its adulterants. In addition, many other plants in *Labiatae* are very similar to MH in traits, such as *Elsholtzia haichowensis* (*E. haichowensis*), *Elsholtzia densa*, *Origanum vulgare*, etc. These plants often appear to be counterfeits of MH in the market. Even in the medicinal history of MH, there has been an embarrassing situation for many years, especially when *E. haichowensis* has been mistaken as an authentic product of MH. Naturally, the clinical efficacy of MH has been challenged due to the different chemical compositions and biological activities caused by the growth environment, harvest time, and species differences. Most of the early research on MH focused on the field of botany, which mainly involved the extraction and identification of compounds in plants. With the continuous updating of research methods and techniques, research on the pharmacological effects of MH is also widely carried out. Subsequently, Chinese patent medicines represented by Shure Ganmao Keli and Xiangju Ganmao Keli were produced. These products are widely sold in China for their good efficacy in treating colds. Therefore, this paper highlights the importance of accurate identification of MH medicinal plants. The research progress of MH in botany, traditional use, phytochemistry, and pharmacological activity is summarized. The research direction of MH is also prospected. It is hoped that these works can provide a broader background and ideas for future research on MH. And then enhance the potential impact of future MH research on public health or the pharmaceutical industry. The known components and pharmacological effects of MH are shown in Figure 1.

## 2. Botany

There are many records about MH in the works of traditional Chinese medicine, and its dual use of medicine and food can be traced back to 1500 years ago. Today, it is also one of the few medicinal and food varieties approved by the Ministry of Health of China.

*M. chinensis* Maxim and *M. chinensis* ‘Jiangxiangru’ have strong adaptability. They all like to grow in warm, humid, and sunny environments. The superior geographical conditions in Jiangxi are more in line with the growth needs of *M. chinensis* ‘Jiangxiangru’. At present, the cultivation and planting of *M. chinensis* ‘Jiangxiangru’ in this area have been scaled and industrialized. The planting area has exceeded 33.3 hectares [17]. The suitable sowing time for *M. chinensis* ‘Jiangxiangru’ is from late March to mid-April. The best sowing method for easy management is strip sowing. After sowing, cover about 1 cm thick with plant ash or fire ash. The growth cycle of *M. chinensis* ‘Jiangxiangru’ is short. After emergence, seedlings should be fixed in a timely manner. After that, it is necessary to fertilize and weed in time and control irrigation and drainage. From mid to late August, when *M. chinensis* ‘Jiangxiangru’ grows to the flowering period, it is harvested. The medicinal herbs with thick leaves, rich aroma, and many spikes are preferred [18]. Then, this article comprehensively references the two drug standards of Chinese pharmacopoeia and the standard compilation of genuine medicinal materials [19]. The differences between the two MHs were discussed in detail from the perspective of medicinal materials and plants. It is hoped to provide an effective theoretical basis for the application and identification of MH.

*M. chinensis* Maxim is an annual herb, mostly wild, with a strong fragrance. It often grows on the hillside or under the forest at an altitude of 1400 m and is mainly located in Anhui, Jiangsu, Guangdong, Guangxi, Hunan, and Hubei in China. It is 30–50 cm long, with a purple-red base and gray-green upper part, and is densely covered with white hairs. Stem square cylindrical, base nearly round, diameter 1–2 mm, obvious nodes, internode length 4–7 cm; brittle and easy to break. Leaves opposite, much wrinkled or abscisic, leaf blade long ovate or lanceolate after spreading, dark green or yellowish green, margin 3 to 5 sparsely serrate. Spikes terminal and axillary, bracts ovate or obovate, falling off or remaining; calyx persistent, campanulate, light purple red or gray-green, apex 5-lobed, densely pubescent. The nutlets are nearly subglobose, with a diameter of 0.7–1.1 mm, and reticulated. The upper epidermal cells of *M. chinensis* Maxim leaves were polygonal; the anticlinal wall was wavy, curved, and slightly thickened. The cell wall of the lower epidermis is not thickened, and the stomata is on a straight axis. The glandular scale has eight cells in its head, with a diameter of about 36–80 μm. The upper and lower epidermis have non-glandular hairs, which are mostly broken, and the upper cells are mostly bent in a hook shape, with obvious warty protrusions.

*M. chinensis* ‘Jiangxiangru’ is an artificially planted variety. Fenyi County, Jiangxi Province, China, is its proper producing area. The cultivated *M. chinensis* ‘Jiangxiangru’ has large quantity and high quality, which can be widely sold and applied. *M. chinensis* ‘Jiangxiangru’ is 55–66 cm long, with a yellow-green surface and a relatively soft texture. The edges have 5–9 sparse shallow serrations. The fruit is 0.9–1.4 mm in diameter with a sparse reticulate surface. The upper epidermal glandular scale of the leaves is about 90 μm in diameter; the non-glandular hair is mostly composed of 2–3 cells. The lower cells are longer than the upper cells, and the warty protrusions are not obvious. Non-glandular hair basal podocytes 5–6, and the anticlinal wall is beaded thickening. The summary of MH botany is given in Table 1.

## 3. Traditional Uses

The *Mosla* species can be classified into three categories based on their traditional uses: medicine, dietary health care, and ornamental plants. Their use as medicinal plants dates back more than 400 years to the “Compendium of Materia Medica”. However, only a few species of this genus have been recorded in traditional records. Chinese pharmacopoeia also included only *M. chinensis* Maxim and *M. chinensis* ‘Jiangxiangru’, with a recommended dosage of 3–10 g. Regarding the traditional application of MH, the main diseases treated by MH alone include cholera, nosebleeds, beriberi, halitosis, and edema. Compound medication mainly uses Xiangru San to treat heatstroke, diarrhea, typhoid fever, etc. [20]. MH is generally not used as a single drug in modern clinical practice and often exists in the form of prescriptions, such as Xiangru San, Qinghao Xiangru San, Xinjia Xiangru Yin, and Chaihu Xiangru Yin. On the market, there are Chinese patent medicines made of capsules, granules, tablets, pills, and other dosage forms made of volatile oils and extracts of MH. Moreover, modern doctors are mostly based on Xinjia Xiangru Yin, and the clinical application is more and more flexible with the addition and reduction of the syndrome, which can be used to treat common cold due to summer heat and dampness [21], acute pharyngeal conjunctival fever in children [22], acute upper respiratory infection in summer [23], etc. In addition, the stems and leaves of MH are fresh, fragrant, and sweet, commonly used as vegetables. The porridge cooked with MH has the effects of sweating, relieving external symptoms, eliminating heat and dampness, and promoting water and swelling. MH can also be used as a medicinal tea, such as Xiangru Bohe tea and Xiangru Erdou Yin. When boiling tea, mint and light bamboo leaves are added at the same time to play the role of clearing heat, removing trouble, diuresis, and clearing heart. The traditional uses of MH are summarized in Table 2.

## 4. Phytochemistry

**Figure 2:**
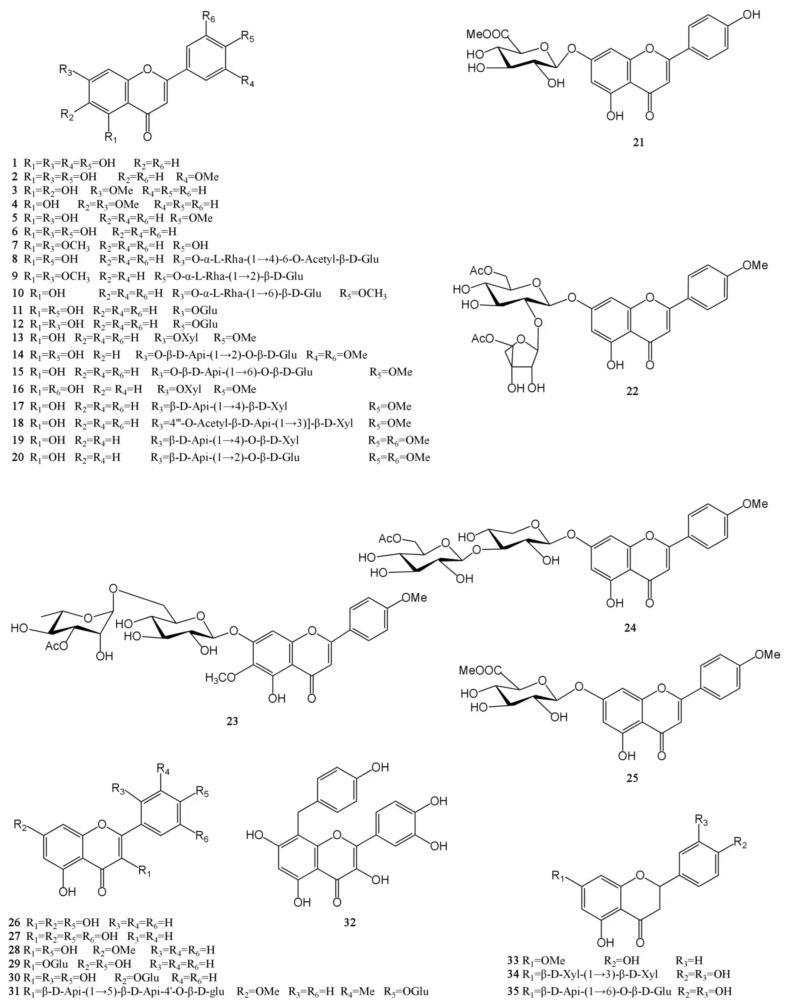
Structures of flavonoids isolated from the MH.

**Figure 3:**
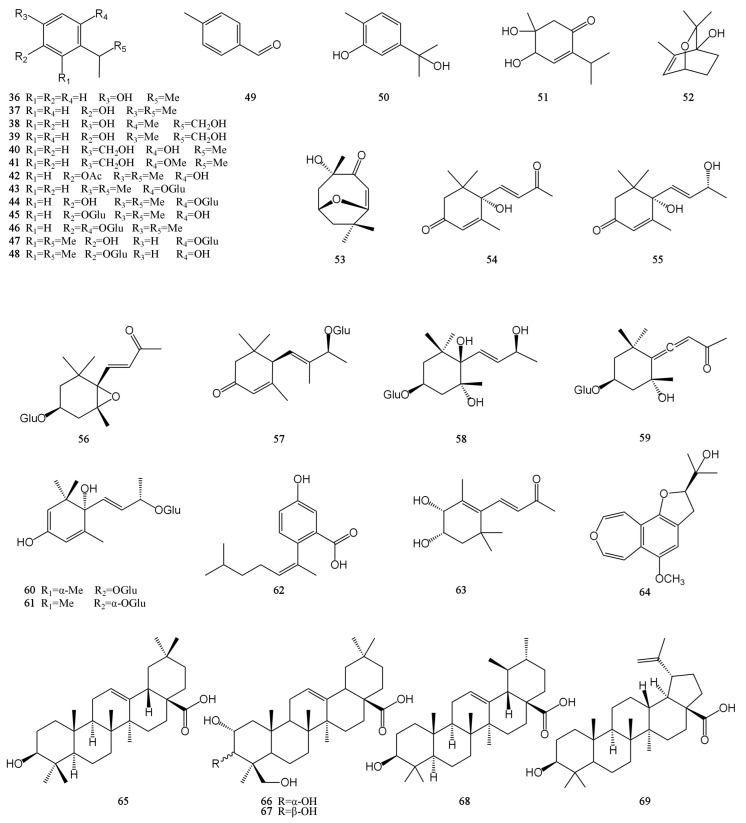
Structures of terpenes isolated from the MH.

**Figure 4:**
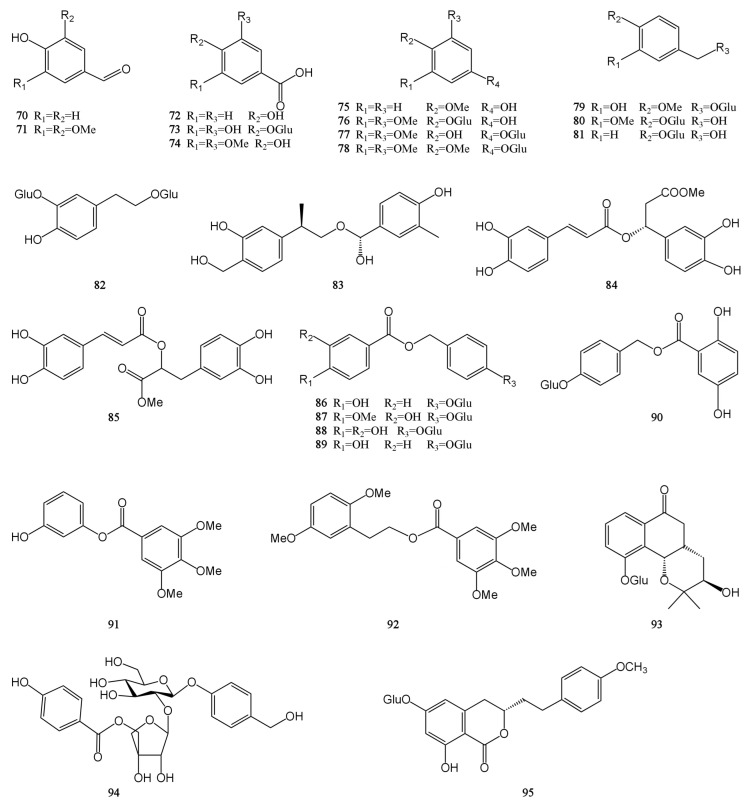
Structures of phenolic acids isolated from the MH.

**Figure 5:**
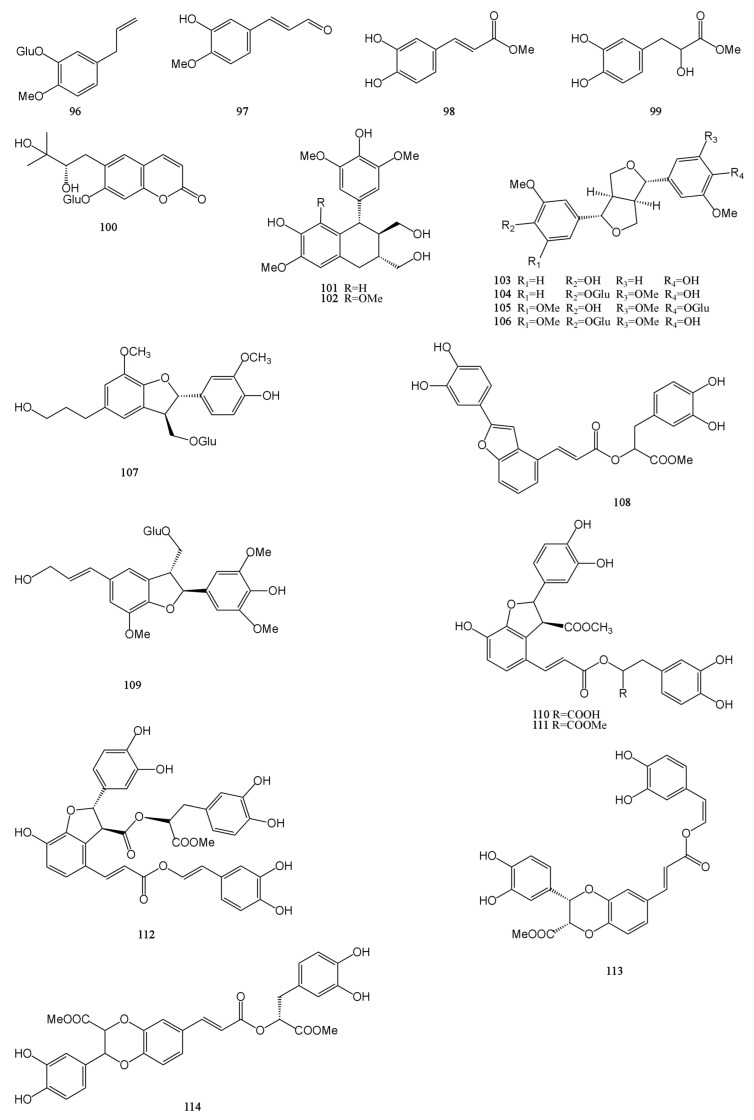
Structures of phenylpropanoids isolated from the MH.

**Figure 6:**
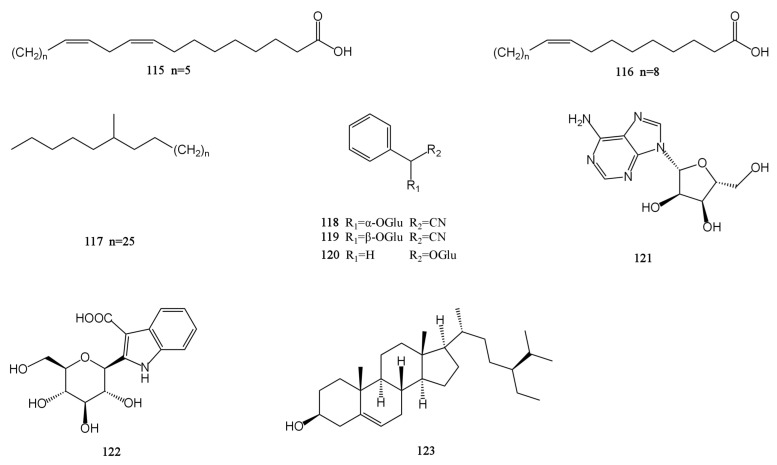
Structures of others isolated from the MH.

At present, approximately 123 compounds have been isolated and identified from MH, including flavonoids (**1**–**35**), terpenoids (**36**–**69**), phenolic acids (**70**–**95**), phenylpropanoids (**96**–**114**), and others (**115**–**123**). Among them, there are more flavonoids and terpenoids, which play a direct or indirect role in the pharmacological activity of MH. In addition, MH is rich in volatile oils, and according to the available literature data, it is found to contain a large number of bioactive components. The isolated compounds are listed in Table 3. Their chemical structures are shown in Figure 2, Figure 3, Figure 4, Figure 5 and Figure 6.

### 4.1. Volatile Oils

The plants of the genus Mosla are generally aromatic and rich in volatile oils. Their extraction methods are generally steam distillation and supercritical CO_2_ extraction. The extraction rate of the volatile oils extracted using the previous method after purification is 0.5% (*v*/*w*). The volatile oils of *M. chinensis* Maxim and *M. chinensis* ‘Jiangxiangru’ are mainly composed of monoterpenoids and sesquiterpenoids. The monoterpenoids in *M. chinensis* ‘Jiangxiangru’ volatile oils are more than those in *M. chinensis* Maxim, while the sesquiterpenoid compounds in *M. chinensis* Maxim are much more than those in *M. chinensis* ‘Jiangxiangru’. The volatile oils of *M. chinensis* Maxim are mainly composed of terpenoids and alcohols. The main compounds of *M. chinensis* ‘Jiangxiangru’ are mainly phenolic and olefin compounds. Although the components of these two volatile oils are different, their main compounds are thymol and carvacrol (**37**). The content of both can account for more than 50% of the total volatile oils [35,36,37,38]. Due to this, thymol and carvacrol are used as chemical markers for quality control of MH. Now, the research on the volatile oils components of MH is relatively systematic and comprehensive. More than 200 volatile compounds have been analyzed by GC-MS.

### 4.2. Flavonoids

Flavonoids are a widely occurring secondary metabolite in plants, found in almost all green higher plants. The MH flavonoids can be extracted by refluxing with 60% ethanol. Using this method, 3.7% of total flavonoids can be obtained in *M. chinensis* Maxim, with a content of 75.1%. The levels of its main components, luteolin, and apigenin, are 9.57 and 1.05 mg/g, respectively [39]. A total of 35 flavonoids have been isolated from MH [8,24,25,26,27,28,29]. Among them, there are 25 flavones (**1**–**25**), 7 flavonols (**26**–**32**) and 3 dihydroflavones (**33**–**35**). Except for 13 compounds such as luteolin (**1**), chrysoeriol (**2**), and negletein (**3**), which were found in the form of aglycones, all the other flavonoids were found in the form of glycosides and were mainly linked to sugars and their derivatives at C_5_ and C_7_ positions. Moreover, all flavonoid glycosides obtained in MH are *O*-glycosides, and aglycones mainly include apigenin, acacetin, luteolin, etc. [24,28]. Flavonoids have a wide range of biological activities, and they have important applications in new drug development, food storage, the cosmetics industry, and other fields. Luteolin has been shown to have antioxidant, anti-inflammatory, antimicrobial, and anticancer activities and has the potential to treat respiratory diseases, which has attracted much attention [40,41]. In addition, the quercetin (**27**) has poor water solubility and low bioavailability. In order to exert its biological activity, the research on quercetin and its derivatives is increasing at home and abroad. It mainly involves the modification of the quercetin skeleton and the esterification, amination, and sulfation of quercetin. The quercetin-3-*O*-amide derivatives were obtained by a series of reactions such as benzyl selective protection and Williamson ether formation of quercetin. This new quercetin derivative has better antitumor activity than the parent drug quercetin [42]. The chemical structures of these molecules are presented in Figure 2.

### 4.3. Terpenoids

Terpenoids are polymers of isoprene and its derivatives. They are a class of natural products with a wide variety and complex structures in nature. Up to now, 34 terpenoids have been isolated from MH, including 18 monoterpenes (**36**–**53**), 11 sesquiterpenes (**54**–**64**), and 5 pentacyclic triterpenoids (**65**–**69**), and all monoterpenes and sesquiterpenes are monocyclic [7,26,28,30,31,32,33,34]. Except for 4,5-dihydroxy-5-methyl-2-(1-methylethyl)-2-cyclohexen-1-one (**51**), all monocyclic monoterpenes have a benzene ring structure. However, only five pentacyclic triterpenoids are known from MH, including three oleanane-type (**65**–**67**), one ursane-type (**68**), and one lupine-type (**69**) pentacyclic triterpenoids. The carvacrol is easy to pass through the cell membrane because of its small molecular weight and high-fat solubility. At the same time, it also exhibits a certain degree of hydrophilicity due to the presence of phenolic hydroxyl groups. The hydroxy group of carvacrol can be prenylated to form a lipophilic prodrug of carvacrol. This synthesized compound improved the membrane permeability and oral absorption rate of carvacrol. It can exist stably in human plasma without cytotoxicity [43]. In addition to preventing and treating diseases, some terpenoids in MH can also be used as food seasonings and preservatives within a certain concentration range, such as thymol. Their chemical structures are shown in Figure 3.

### 4.4. Phenolic Acids

Phenolic acids generally refer to a class of compounds with several hydroxyl groups on the same benzene ring, and there are mainly two carbon skeleton types: benzoic acid type (C_6_–C_1_) and cinnamic acid type (C_6_–C_3_). In addition to these two phenolic acids, most phenolic acids can generate complex phenolic acid derivatives due to the interaction between active groups. And these complex derivatives include gallic acid derivatives, phloroglucinols, salvianolic acids, chlorogenic acids, etc. The study found that MH contains 26 phenolic acids, mainly benzoic acid type. They mostly exist in the form of amides, esters, or glycosides and rarely exist in free form [7,9,28,30,31,32]. Syringic acid (**74**) is biosynthesized in plants through the shikimic acid pathway and is commonly found in Leonurus japonicus and Dendrobium nobile. It is reported to have antioxidant, anti-inflammatory, lipid-lowering, nerve, and liver protection activities. In addition, it has shown a wide range of therapeutic applications in the prevention of diabetes, cardiovascular disease, cancer, and cerebral ischemia [44]. Gastrodin (**81**) is a small molecule active compound isolated from the tubers of Gastrodia elata. It has a strong neuroprotective effect and can improve myocardial hypertrophy, hypertension, and myocardial ischemia-reperfusion injury [45]. The chemical structures of compounds 70–95 are shown in Figure 4.

### 4.5. Phenylpropanoids

Phenylpropanoids are a group of natural organic compounds containing one or more C_6_–C_3_ structures. A total of 19 phenylpropanoids were obtained and identified from MH [7,9,24,29,31,32,33], which are mainly divided into three categories: simple phenylpropanoids (**96**–**99**), coumarins (**100**), and lignans (**101**–**114**). Among them, lignans accounted for a higher proportion, a total of 14. Dimethyl clinopodic acid C (**114**) is a new neolignan whose parent core is a benzodioxane ring and which also has the structure of phenol. Isoeucommin A (**104**) is a lignan isolated from Eucommia ulmoides, which can reduce renal injury by activating nuclear factor erythroid2-related factor 2/heme oxygenase-1 (Nrf2/HO-1) signaling pathway to reduce inflammation and oxidative stress [46]. Lyoniresinol (**102**) has antioxidant and cell protective activities and can reduce middle cerebral artery occlusion brain ischemic injury in rats by inhibiting oxidative stress [47]. Their chemical structures are described in Figure 5.

### 4.6. Others

There are nine other compounds in total, including two fatty acids (**115**–**116**), one alkane (**117**), three glycosides (**118**–**120**), one nucleoside (**121**), one alkaloid (**122**), and one steroid (**123**) compound [26,28,30,31,32,33]. The β-sitosterol (sit) is a white crystalline substance. It is structurally very similar to cholesterol. The researchers obtained a new ester derivative (Sit-S) by esterifying and derivating it. This compound is more fat-soluble and can further enhance the antidepressant effect of β-sitosterol [48]. There are also polysaccharides in MH. The polysaccharide hydrolytic derivative of *M. chinensis* ‘Jiangxiangru’ is analyzed by gas chromatography, and it is found that the crude polysaccharide CMP (CMP) of *M. chinensis* ‘Jiangxiangru’ is composed of rhamnose, ribose, fucose, arabinose, xylose, mannose, glucose, and galactose. Refined polysaccharide MP (MP) is less ribose and fucose in composition than CMP. The neutral polysaccharide MP-1 (MP-1) is composed of arabinose, rhamnose, mannose, glucose, galactose, and trace fucose. The neutral part of the acid polysaccharide MP-A40 (MP-A40) of *M. chinensis* ‘Jiangxiangru’ is composed of rhamnose, arabinose, glucose, mannose, and galactose, and the authors confirmed that the polysaccharide of *M. chinensis* ‘Jiangxiangru’ has certain antioxidant and immune regulation effects [49,50]. In addition, *M. chinensis* ‘Jiangxiangru’ also contains 25 mineral elements, including K, Ca, P, Mn, Fe, etc. [51]. The chemical structures of compounds 115–123 are presented in Figure 6.

## 5. Pharmacological Activities

**Figure 7:**
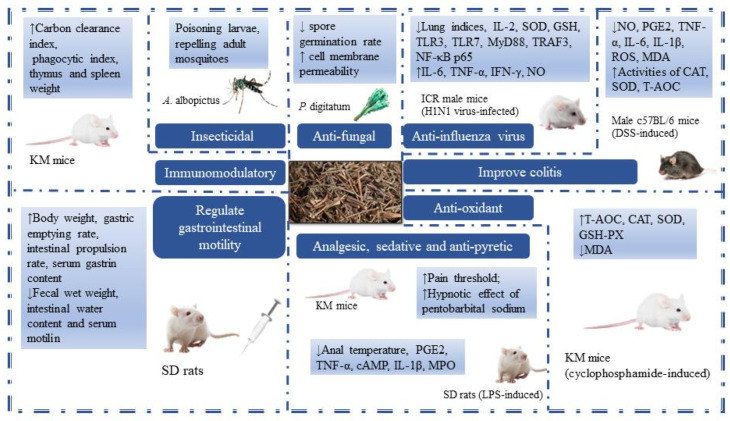
The pharmacological activities of MH (Arrow ↓ means decrease, Arrow ↑ means increase.)

TCM theory believes that MH has the functions of sweating and relieving the exterior, resolving dampness, and eliminating heat, so it is also called the “Xiayue Zhi Mahuang”. MH has antibacterial, antiviral, anti-inflammatory, antioxidant, analgesic sedation, antipyretic, and immune-enhancing effects and is widely used in clinical practice. In the early stage, the research on MH chemical components was limited to the extraction and separation of its main component, volatile oils, and the research on its pharmacological effects mainly focused on the volatile oils. Therefore, the chemical constituents and pharmacological activities of MH, in addition to volatile oils, still need to be further studied. However, due to the experimental environment and some human factors, the experimental results may have some differences. In order to make a more comprehensive evaluation of MH, we still summarized the reports on MH as much as possible. It is hoped that this will increase the objectivity and authenticity of MH evaluation. The pharmacological activities of MH are summarized in Table 4 and Figure 7.

### 5.1. Antibacterial and Antiviral

MH volatile oils have strong broad-spectrum antibacterial ability. It has been reported that MCM volatile oil and MCJ ethyl acetate extract have inhibitory effects on common bacteria such as *Staphylococcus aureus* and *Escherichia coli* [52,66]. The researchers evaluated the inhibitory effect of the active ingredient carvacrol in *M. chinensis* ‘Jiangxiangru’ on *Penicillium digitatum* (*P. digitatum*). The results showed that the minimum inhibitory concentration (MIC) and minimum bactericidal concentration (MFC) of carvacrol against *P. digitatum* were 0.1 mg/mL and 0.2 mg/mL, respectively. The changes in *P. digitatum* treated with carvacrol were obvious, mainly manifested as a significant decrease in spore germination rate, an increase in cell membrane permeability, and a decrease in soluble sugar content in the bacteria [67]. The above shows that carvacrol can achieve a certain antibacterial effect by affecting the normal growth and development of bacteria. In terms of antiviral, the volatile oils from *M. chinensis* Maxim had certain antagonistic effects on Newcastle disease virus (NDV), and 0.3 g/L and 0.7 g/L volatile oils from *M. chinensis* Maxim had stronger anti-NDV effects than 1.0 g/L ribavirin. The antiviral effect of volatile oils increased with the increase of drug concentration within the safe dose range [53].

MH has been a typical TCM for the treatment of summer colds since ancient times. Modern pharmacological studies have also found that MH has a strong anti-influenza virus effect. For example, *M. chinensis* Maxim volatile oils could effectively inhibit the cytopathic effect (CPE) of Vero cells caused by the A3 virus. Using lung index as an indicator, it was found that when the dose was above 100 μg/(g·d), the virus-induced pneumonia in mice was significantly inhibited [54]. Except for volatile oils, *M. chinensis* Maxim total flavonoids also have a certain clearance rate against the H1N1 influenza virus. And it can reduce inflammatory lung tissue injury by regulating inflammatory cytokines and antioxidant factors (such as IL-6, TNF-α, SOD, and GSH) [39]. For compounds 3-(3,4-dihydroxyphenyl) acrylic acid 1-(3,4-dihydroxyphenyl)-2-methoxycarbonylethyl ester (**84**) and monomethyl lithospermate (**110**) isolated from *M. chinensis* Maxim 70% acetone extract, the researchers found that when the screening concentration reached 100 μmol/L, the inhibition rates of the two compounds against H1N1 influenza virus reached 89.3% and 98.6%, respectively [9]. MH’s existing products for the treatment of colds, such as ShuReGanMaoKeLi and XiangRuWan, are mainly composed of a variety of drugs. Clarifying the mechanism of MH treatment against the influenza virus is of great significance for MH to give full play to its drug effect while simplifying its prescription.

### 5.2. Anti-Inflammatory

MH can improve some inflammatory reactions. For example, the water extract of *M. chinensis* Maxim could inhibit mast cell-mediated allergic inflammation and allergic reactions caused by compound **48**/**80** and immunoglobulin E (IgE). In addition, *M. chinensis* Maxim reduced gene expression and secretion of proinflammatory cytokines such as tumor necrosis factor (TNF)-α, interleukin (IL)-6, and IL-8 in human mast cells [55]. The results provided a possibility for MH to be used as a drug to prevent or treat allergic inflammatory diseases mediated by mast cells. *M. chinensis* ‘Jiangxiangru’ methanol extract reduced the production of reactive oxygen species (ROS) in a 3% sodium gluconate-induced colitis model mice and increased the activity of the antioxidative enzyme. Meanwhile, the secretion of inflammatory mediators (NO, PGE2) and cytokines (TNF-α, IL-6, IL-1β) in mice was inhibited. The activation of mitogen-activated protein kinases (MAPKs) signaling pathway was also inhibited. Furthermore, compared with the model group, the tissue damage in the *M. chinensis* ‘Jiangxiangru’ treatment group was lighter, and the histological score was lower [56]. Under the intervention of *M. chinensis* Maxim volatile oils, pro-inflammatory factors (TNF-α, IL-1β, IL-6) in the serum of mice with lipopolysaccharide (LPS) induced showed a certain downward trend, while anti-inflammatory factors (IL-10) showed a certain upward trend. When the dose reached 0.2 mL/kg, the anti-diarrhea ability of *M. chinensis* Maxim volatile oils was basically the same as that of 100 mg/kg chlortetracycline. The mechanism might be through down-regulating the expression of the TLR4/NF-κB signaling axis and up-regulating the expression of intestinal tight junction proteins, thereby reducing intestinal damage [57].

### 5.3. Antioxidant

The antioxidant activity of *M. chinensis* Maxim was determined by DPPH assay, β-Carotene bleaching assay, reductive potential assay, and total phenol content determination. It was found that the *M. chinensis* Maxim volatile oils were greater than its methanol extract [68]. The DPPH, ABTS, and FRAP methods were also used to screen the antioxidant activity of *M. chinensis* ‘Jiangxiangru’ active components extracted by five different solvents. It was found that their activity sequence is 70% ethanol extract > anhydrous ethanol extract > 30% ethanol extract > water extract > chloroform extract [38]. Another study evaluated the antioxidant activity of the total flavonoids of *M. chinensis* Maxim using DPPH, pyrogallol autoxidation, and FRAP method. It was found that the total flavonoids of *M. chinensis* Maxim had good scavenging ability for DPPH free radicals and O^2−^ and a strong reducing ability for Fe^2+^. When the total flavonoid concentration was 143 μg/mL, its reduction ability of Fe^3+^ was approximately equivalent to that of 69.7 μg/mL vitamin C solution [69]. The polysaccharide MP isolated from *M. chinensis* ‘Jiangxiangru’ could significantly increase the levels of T-AOC, CAT, SOD, and GSH-PX and reduce the level of MDA in immunosuppressed mice induced by cyclophosphamide (CTX). And the chelating ability of MP-1 and ferrous ions was dose-dependently enhanced. When the concentration of MP-1 increased from 0.5 mg/mL to 16 mg/mL, the chelation rate of ferrous ions increased from 7.1% to 87.8% [50,62]. Similar results were obtained in studies of other *M. chinensis* ‘Jiangxiangru’ polysaccharides. The acidic components JXR-1 and JXR-2 showed scavenging activity on hydroxyl radicals and superoxide anion radicals and showed a certain total reduction ability [70].

### 5.4. Analgesic, Sedative and Antipyretic

Both *M. chinensis* Maxim and *M. chinensis* ‘Jiangxiangru’ volatile oils could increase the pain threshold of mice, and there was a dose–effect relationship between 0.1 and 0.3 mg/kg. The analgesic effect of volatile oils of *M. chinensis* ‘Jiangxiangru’ was stronger than that of *M. chinensis* Maxim at the same dose. Furthermore, when the dose was 0.3 mL/kg, the analgesic effect of the two was more lasting than that of 30 mL/kg tetrahydropalmatine sulfate [58]. The different concentrations of *M. chinensis* Maxim and *M. chinensis* ‘Jiangxiangru’ volatile oils were given to mice. The results showed that when the dose was greater than 0.1 mL/kg, both of them could inhibit the writhing response induced by acetic acid in mice and enhance the hypnotic effect of pentobarbital sodium [59]. *M. chinensis* Maxim, based on integration processing technology of origin, can reduce the body temperature of fever rats induced by LPS. Compared with aspirin, MH water extract and volatile oils had a faster antipyretic effect, but the effect did not last long. There was a significant dose–effect relationship between the antipyretic effects of MH volatile oils and water extract. Within 350 min after administration, MH water extract took effect faster and had a longer duration of action than MH volatile oils [12]. The research has shown that *M. chinensis* Maxim and *M. chinensis* ‘Jiangxiangru’ volatile oils can reduce the body temperature of normal mice and have antipyretic effects on yeast-induced fever rats. When the dose was approximately 0.1 mL/kg, *M. chinensis* Maxim had a stronger cooling effect than *M. chinensis* ‘Jiangxiangru’. Whether it was *M. chinensis* Maxim or *M. chinensis* ‘Jiangxiangru’, the action time of a high dose was greater than that of a low dose [58]. In conclusion, MH does have anti-influenza and antipyretic effects, but whether there is a certain relationship between these two effects and whether MH can alleviate the symptoms of fever caused by colds has not been scientifically confirmed.

### 5.5. Regulate Gastrointestinal Motility

Nowadays, patients with gastrointestinal diseases account for a high proportion of the global population, and the number of patients is increasing year by year. The three countries with the highest incidence of gastrointestinal diseases in the world are China, Japan, and South Korea. Gastrointestinal diseases are generally difficult to cure and easy to relapse. Finding suitable drugs to treat such diseases will greatly improve the happiness index of human life. The studies have shown that the volatile oils of *M. chinensis* Maxim and *M. chinensis* ‘Jiangxiangru’ had a significant antagonistic effect on the contraction caused by acetylcholine. The effect of *M. chinensis* ‘Jiangxiangru’ was stronger than that of *M. chinensis* Maxim [59]. *M. chinensis* Maxim volatile oils could also effectively regulate water metabolism and had a good effect on resolving dampness and invigorating the spleen [60]. Additionally, *M. chinensis* Maxim volatile oils had a dual regulatory effect on gastrointestinal motility in mice. The concentration of 0.1‰ of volatile oils had a significant inhibitory effect on the normal rhythmic contraction of the isolated duodenum in mice, while the concentration from 0.03‰ to 0.06‰ showed a promoting effect. Moreover, the regulatory effect of volatile oils on the tense contraction of the duodenum only changed the contraction amplitude and did not change the contraction frequency [61].

### 5.6. Immunomodulatory

Immunity is a physiological protective function of the body. The volatile oils of *M. chinensis* Maxim could significantly increase the carbon particle clearance index and phagocytosis index of normal mice, increase the weight of the thymus and spleen, and enhance delayed-type hypersensitivity (DTH) of mice skin caused by 2,4-dinitrofluorobenzene (DNFB) [13]. These results indicated that *M. chinensis* Maxim volatile oils could enhance both specific and non-specific immunity in normal mice. In the study of MP with low protein content in *M. chinensis* ‘Jiangxiangru’, it was found that MP and CTX increased thymus and spleen indexes compared with the negative group. After three days of single administration of CTX, the immune function of mice decreased. The above indicated that MP can, to some extent, overcome the immunosuppressive effect of CTX [62]. In addition to MP, other polysaccharide fractions isolated from *M. chinensis* ‘Jiangxiangru’ also had immunomodulatory effects. For example, MP-1 promoted the proliferation of T-cells induced by concanavalin A (ConA) and B cells induced by LPS. And it was more effective in promoting ConA-induced T-cell proliferation [50]. The MP-A40 enhanced the production of NO in RAW264.7 macrophages in a dose-dependent manner. When the concentration of MP-A40 was 10 μg/mL, the production of NO was about 15 times that of the negative control [71].

### 5.7. Insecticidal

The bioactive substances extracted from plants have been proven to be useful for pest control, including alkaloids, glycosides, tannins, terpenoids, volatile oils, and other compounds. The main compounds of MH volatile oils are monoterpenoids, sesquiterpenoids, and aromatic hydrocarbon derivatives, which have the effects of repelling, inhibiting growth, and being toxic to pests. The researchers compared the morphological changes of trichomonas vaginalis (TV) after in vitro giving *M. chinensis* Maxim, *Zanthoxylum bungeanum* Maxim, and *Dichroa febrifurga* Lour aqueous extracts. The results showed that *M. chinensis* Maxim had the best insecticidal effect, and the contents of TV were spilled and decomposed into fragments after *M. chinensis* Maxim treatment [63]. In the fumigation experiment, the mortality of both adults and nymphs of *Aphis gossypii* (*A. gossypii*) treated with *M. chinensis* Maxim volatile oils could reach more than 85%. It can be seen that *M. chinensis* Maxim volatile oils have a strong killing effect on adult and instar nymphs of *A. gossypii* [14]. The biological activity of *M. chinensis* Maxim volatile oils against *Aedes albopictus* (*A. albopictus*) was determined. It was found that the half-lethal concentrations (LC_50_) of *M. chinensis* Maxim volatile oils against larvae and pupae of *A. albopictus* were 78.8 and 122.6 μg/mL, respectively. After the local application of 1.5 mg/cm^2^ pure volatile oils on the human body, the complete protection time for adult mosquitoes was 2.3 h, and the protection rate was still 56% after 6 h [64]. This provided a theoretical basis for the further development of new mosquito control agents and insecticides using the volatile oils of *M. chinensis* Maxim. All in all, we can continue to explore the toxic effect of MH volatile oils on pests, find its insecticidal mechanism and target, and develop new products with higher pest control and insecticidal ability.

### 5.8. Others

The α-glucosidase is involved in the digestion and absorption of carbohydrates, starch, and glycoproteins. It can inhibit the decomposition of α-glucosidase and carbohydrates, reduce the production and absorption of glucose, and then control postprandial blood glucose. At present, α-glucosidase inhibitors have been recognized as the main treatment for type 2 diabetes. It has been reported that *M. chinensis* ‘Jiangxiangru’ can inhibit α-glucosidase activity to a certain extent. The effects of volatile oils and 75% ethanol extract ethyl acetate extract layer were better. When the concentration of ethyl acetate layer was 1.0 mg/mL, the inhibition rate reached 84.2%. At the concentration of 0.6 μL/mL, the inhibition rate of the volatile oils prepared by steam distillation was close to 100% [65]. Therefore, MH can be used as a potential resource for natural α-glucosidase inhibitors and has high research value.

The MP-A40 had an obvious inhibitory effect on the proliferation of human chronic myeloid leukemia cells (K562 cells), and the inhibitory effect was enhanced with the increase of the concentration of MP-A40 [71]. This indicated that MP-A40 has the ability to inhibit the proliferation of tumor cells in vitro. At the same time, this was also the first time a component with the ability to inhibit tumor cells in MH was found nationally and internationally. The processing of MH is relatively simple, and most of them are cut directly after drying. This may be due to the loss of MH volatile oils during processing, which affects clinical efficacy. Correspondingly, there are few studies on the processing of MH and only reports that MH processed with ginger juice can enhance the sweating and hemostatic abilities of mice [72,73]. In addition, *M. chinensis* Maxim had a good fresh-keeping effect on meat [74]. Because of its pure fragrance and antibacterial, it could be used as a tobacco essence [75], air freshener [76], etc. Therefore, we can also try to process MH into people’s favorite perfume within a safe range so that MH resources can be fully utilized.

## 6. Conclusions and Prospect

MH, as a natural food with development value, also has a wide range of biological activities. It has a long history of medication in China, and many ancient books have recorded its medicinal value. This paper mainly reviews the research results of MH in botany, traditional application, phytochemistry, and pharmacology in recent years. And summarizes the structures of more than 100 compounds isolated from them. Furthermore, the pharmacology section also lists the research of MH in antibacterial, antiviral, anti-inflammatory, antioxidant, analgesia, sedation, antipyretic, regulation of gastrointestinal movement, regulation of immunity, insecticidal and other aspects.

Based on previous studies, we summarized 123 compounds. Some of these compounds are used as indicators to identify the authenticity of MH and evaluate the quality of MH. For example, the Chinese Pharmacopoeia stipulates that thymol and carvacrol are used as reference substances to determine the authenticity of MH by thin-layer chromatography. However, using only the total volatile oil and its main compound content as the quality standard for MH is relatively single. On this basis, other indicators can be considered to establish a comprehensive quality control and evaluation system. To ensure the safety and effectiveness of MH clinical medication.

MH was mainly used to treat summer colds in the past. Modern pharmacological studies have shown that it can fight against H1N1 and A3 viruses and has the potential to treat influenza. This finding is consistent with the traditional sweating and surface-relieving effects of MH. The pharmacological activity for the treatment of influenza was again confirmed. In summary, we can make clear that MH has a certain scientific connotation in the treatment of summer-damp cold. At the same time, exploring the possible indications of MH from traditional applications also provides some ideas for its modern development.

There are many traditional chemical pesticide products, including organochlorines, organophosphorus, and carbamates. While killing insects, these products also have some negative effects that cannot be ignored, such as DDT and Temik. Previous studies have found that MH volatile oils have a good insecticidal effect. MH can be used as a raw material for natural insecticides to develop green and environmentally friendly botanical insecticide products.

Although the research on MH has achieved certain results, there are still some problems that need to be addressed. Firstly, the existing data show that compared with other components, the study of volatile oil is more in-depth. Further exploration of other components and their biological activities in MH is needed. Secondly, Chinese medicine processing is a traditional pharmaceutical technology in China. In the Ming Dynasty, there was a method of processing MH with ginger juice. Modern pharmacological studies have found that processed MH has a stronger sweating effect. This may be due to a certain synergistic effect between ginger and MH. However, the mechanism, target, and pathway of action of ginger MH have not been scientifically elucidated. Whether ginger products have other pharmacological activities is unclear. In conclusion, based on the characteristics of MH dual-use of medicine and food, it has shown great development and utilization value in food processing, health care, and cosmetics industries. In this paper, the research progress of MH is reviewed in order to provide a basis and ideas for its future research, development, and application.

## Figures and Tables

**Table 1:** Summary of MH botany.

Items	*M. chinensis* Maxim	*M. chinensis* ‘Jiangxiangru’
Variety	Wild products	Cultivated products
Geographical distribution (global)	Southern China, Northern Vietnam	
Geographical distribution (in China)	Anhui, Jiangsu, Guangdong, and other places	Jiangxi
Planting area (in China)	Unknown	33.3 hectares
Color	Basal purple-red, upper gray-green	Basal purplish red, upper yellowish green or pale yellow
Stem	Upper part is square-cylindrical, branched	Square column, longer
Flower	Many with no flowers	Covered with dense white pubescence
Taste	Spicy and cool with a slight burning sensation	Cool and slightly spicy, slightly numb after
Molecular identification	DNA barcode technology (matK and ITS2 sequences)	

**Table 2:** Summary of the traditional uses of MH.

Items	Traditional Uses		
	Medicine	Dietary Health Care	Ornamental Plants
Usage/dosage	3–10 g	Make porridge and tea	No
Traditional formula/compatibility	Xiangru San, Xiangru Yin	MH with rice, MH with ginger, MH with mint and light bamboo leaves	No
Modern formula/compatibility	Qinghao Xiangru San, Xinjia Xiangru Yin, Chaihu Xiangru Yin	Same as above	No
Efficiency	Sweat and relieve the exterior, remove dampness, and harmonize	Clear heat, remove annoyance, diuresis	Decorate and beautify the environment
Main treatment	Common cold due to summer heat and dampness	No	No
Modern products	Shure Ganmao Keli, Xiangju Ganmao Keli, Qushu Pian	Xiangru porridge, Xiangru Bohe tea, Xiangru Erdou Yin	Potted plants

**Table 3:** Chemical compounds isolated from MH.

No.	Compounds	MF	Source	Part of Plant	Ref.
flavonoids					
**1**	Luteolin	C_15_H_10_O_6_	*M. chinensis* Maxim *M. chinensis* ‘Jiangxiangru’	Above ground part, Whole grass	[8,24]
**2**	Chrysoeriol	C_16_H_12_O_6_	*M. chinensis* ‘Jiangxiangru’	Whole grass	[8]
**3**	Negletein	C_16_H_12_O_5_	*M. chinensis* ‘Jiangxiangru’	Whole grass	[8]
**4**	5-Hydroxy-6,7-dimethoxyflavone	C_17_H_14_O_5_	*M. chinensis* Maxim	Whole grass	[25]
**5**	5,7-Dihydroxy-4′-methoxyflavone	C_16_H_12_O_5_	*M. chinensis* Maxim	Whole grass	[26]
**6**	Apigenin	C_15_H_10_O_5_	*M. chinensis* Maxim *M. chinensis* ‘Jiangxiangru’	Whole grass	[8,26]
**7**	5,7-Dimethoxy-4′-hydroxyflavone	C_17_H_14_O_5_	*M. chinensis* Maxim	Whole grass	[27]
**8**	Apigenin-7-*O*-α-L-rhamnosyl(1→4)-6″-*O*-acetyl-β-D-glucoside	C_29_H_36_O_17_	*M. chinensis* Maxim	Whole grass	[27]
**9**	5,7-Dimethoxy-4′-*O*-α-L-rhamnose(1→2)-β-D-glucoside	C_29_H_34_O_14_	*M. chinensis* Maxim	Whole grass	[27]
**10**	Acacetin-7-*O*-rutinoside	C_28_H_32_O_14_	*M. chinensis* Maxim	Whole grass	[27]
**11**	Apigenin-7-*O*-β-glucoside	C_21_H_20_O_10_	*M. chinensis* Maxim	Above ground part	[24]
**12**	Apigenin-4′-*O*-β-glucoside	C_21_H_20_O_10_	*M. chinensis* Maxim	Above ground part	[24]
**13**	Acacetin-7-*O*-β-D-xylopyranoside	C_21_H_20_O_9_	*M. chinensis* Maxim	Above ground part	[24]
**14**	4′,5,7-Trihydroxy-3′,5′-dimethoxyflavone-7-*O*-[β-D-apiofuranosyl (1‴→2″)]β-D-glucopyranoside	C_28_H_32_O_16_	*M. chinensis* Maxim	Above ground part	[24]
**15**	Acacetin-7-*O*-β-D-apiofuranosyl-(1‴→6″)-*O*-β-D-glucopyranoside	C_27_H_30_O_14_	*M. chinensis* Maxim	Above ground part	[24]
**16**	Diosmetin-7-*O*-β-D-xylopyranoside	C_21_H_20_O_10_	*M. chinensis* Maxim	Above ground part	[24]
**17**	Acacetin-7-*O*-[β-D-apiofuransyl-(1‴→4″)]-β-D-xylopyranoside	C_26_H_28_O_13_	*M. chinensis* Maxim	Above ground part	[24]
**18**	Acacetin-7-*O*-[4‴-*O*-acetyl-β-D-apiofuransyl-(1‴→3″)]-β-D-xylopyranoside	C_28_H_30_O_14_	*M. chinensis* Maxim	Above ground part	[24]
**19**	3′,4′-Dimethoxyluteolin 7-*O*-[β-D-apiofuransyl-(1‴→4″)]-β-D-xylopyranoside	C_27_H_31_O_14_	*M. chinensis* Maxim	Above ground part	[28]
**20**	3′,4′-Dimethoxyluteolin 7-*O*-[β-D-apiofuranosyl-(1‴→2″)]-β-D-glucopyranoside	C_28_H_33_O_15_	*M. chinensis* Maxim	Above ground part	[28]
**21**	Apigenin-7-*O*-β-D-glucuronide methyl ester	C_22_H_20_O_11_	*M. chinensis* Maxim	Above ground part	[24]
**22**	Acacetin-7-*O*-[4‴-*O*-acetyl-β-D-apiofuransyl-(1‴→2″)]-6″-*O*-acetyl-β-D-glucoside	C_31_H_34_O_16_	*M. chinensis* Maxim	Above ground part	[24]
**23**	Isolinariin B	C_30_H_34_O_15_	*M. chinensis* Maxim	Above ground part	[24]
**24**	Acacetin-7-*O*-[6‴-*O*-acetyl-β-D-galactopyranosyl-(1→3)]-β-D-xylopyranoside	C_29_H_32_O_15_	*M. chinensis* Maxim	Above ground part	[28]
**25**	Acacetin-7-*O*-glucuronide methylester	C_22_H_20_O_11_	*M. chinensis* Maxim	Above ground part	[28]
**26**	Kaempferol	C_15_H_10_O_6_	*M. chinensis* ‘Jiangxiangru’	Above ground part	[29]
**27**	Quercetin	C_15_H_10_O_7_	*M. chinensis* ‘Jiangxiangru’	Whole grass	[8]
**28**	Rhamnocitrin	C_16_H_12_O_6_	*M. chinensis* Maxim	Whole grass	[25]
**29**	Kaempferol-3-*O*-β-D-glucoside	C_21_H_20_O_11_	*M. chinensis* Maxim	Whole grass	[26]
**30**	Morin-7-*O*-β-D-glucopyranoside	C_21_H_20_O_12_	*M. chinensis* Maxim	Whole grass	[26]
**31**	Rhamnocitrin-3-*O*-β-D-apiosyl-(1→5)-β-D-apiosyl-4′-*O*-β-D-glucoside	C_33_H_40_O_20_	*M. chinensis* Maxim	Whole grass	[26]
**32**	8-*p*-Hydroxybenzylquercetin	C_22_H_16_O_8_	*M. chinensis* Maxim	Above ground part	[28]
**33**	Sakuranetin	C_16_H_14_O_5_	*M. chinensis* Maxim	Above ground part	[28]
**34**	5-Hydroxy-6-methyl-7-*O*-β-D-pyranxylose-(3→1)-β-D-xylofuran dihydroflavonoid glycosides	C_26_H_30_O_13_	*M. chinensis* Maxim	Whole grass	[26]
**35**	Pyrroside A	C_26_H_30_O_15_	*M. chinensis* Maxim	Above ground part	[24]
terpenoids					
**36**	4-Isopropylphenol	C_9_H_12_O	*M. chinensis* Maxim	Above ground part	[28]
**37**	Carvacrol	C_10_H_14_O	*M. chinensis* ‘Jiangxiangru’	Whole grass	[30]
**38**	4-Hydroxy-β-2-dimethyl-benzeneethanol	C_10_H_14_O_2_	*M. chinensis* Maxim	Above ground part	[7]
**39**	(*R*)-2-(3-hydroxyl-4-methylphenyl)-propan1-ol	C_10_H_14_O_2_	*M. chinensis* Maxim	Above ground part	[28]
**40**	5-Hydroxymethyl-2-isopropylphenol	C_10_H_14_O_2_	*M. chinensis* Maxim	Above ground part	[28]
**41**	(4-Isopropyl-3-methoxyphenyl)-methanol	C_11_H_16_O_2_	*M. chinensis* Maxim	Above ground part	[28]
**42**	1,4-Benzenediol-2-methyl-5-(1-methylethyl)-1-acetate	C_12_H_16_O_3_	*M. chinensis* Maxim	Above ground part	[7]
**43**	Thymol-β-glucoside	C_16_H_24_O_6_	*M. chinensis* Maxim	Above ground part	[7]
**44**	6-Hydroxythymol-3-*O*-β-D-glucopyranoside	C_16_H_24_O_7_	*M. chinensis* Maxim	Stems and leaves	[31]
**45**	6-Hydroxythymol-6-*O*-β-D-glucopyranoside	C_16_H_24_O_7_	*M. chinensis* Maxim	Stems and leaves	[31]
**46**	Thymoquinol-2,5-*O*-β-diglucopyranoside	C_22_H_24_O_12_	*M. chinensis* ‘Jiangxiangru’	Whole grass	[30]
**47**	Thymoquinol-5-*O*-β-glucopyranoside	C_16_H_24_O_7_	*M. chinensis* ‘Jiangxiangru’	Whole grass	[30]
**48**	Thymoquinol-2-*O*-β-glucopyranoside	C_16_H_24_O_7_	*M. chinensis* ‘Jiangxiangru’	Whole grass	[30]
**49**	*p*-Tolualdehyde	C_8_H_8_O	*M. chinensis* Maxim	Above ground part	[7]
**50**	8-Hydroxycarvacrol	C_10_H_14_O_2_	*M. chinensis* Maxim	Above ground part	[28]
**51**	4,5-Dihydroxy-5-methyl-2-(1-methylethyl)-2-cyclohexen-1-one	C_10_H_16_O_3_	*M. chinensis* Maxim	Above ground part	[7]
**52**	(1*R*,4*R*)-3,3,5-trimethyl-2-oxabicyclo-[2.2.2]-oct-5-en-4-ol	C_10_H_16_O_2_	*M. chinensis* Maxim	Above ground part	[7]
**53**	Pubinernoid A	C_11_H_16_O_3_	*M. chinensis* Maxim	Above ground part	[7]
**54**	Dehydrovomifoliol	C_13_H_18_O_3_	*M. chinensis* Maxim	Above ground part	[7]
**55**	Vomifoliol	C_13_H_20_O_3_	*M. chinensis* Maxim	Above ground part	[7]
**56**	Icariside B2	C_19_H_30_O_8_	*M. chinensis* Maxim	Stems and leaves	[31]
**57**	9-Hydroxy-megastigma-4,7-dien-3-one-9-*O*-β-D-glucopyranoside	C_20_H_32_O_7_	*M. chinensis* Maxim	Stems and leaves	[31]
**58**	(3*S*,5*R*,6*R*,7*E*,9*S*)-megastigman-7-ene-3,5,6,9-tetrol-3-*O*-β-D-glucopyranoside	C_19_H_34_O_9_	*M. chinensis* Maxim	Stems and leaves	[31]
**59**	Staphylionoside D	C_19_H_30_O_8_	*M. chinensis* Maxim	Stems and leaves	[31]
**60**	(6*S*,9*R*)-roseoside	C_19_H_30_O_8_	*M. chinensis* ‘Jiangxiangru’	Whole grass	[32]
**61**	Corchoionoside C	C_19_H_30_O_8_	*M. chinensis* Maxim *M. chinensis* ‘Jiangxiangru’	Stems and leaves Whole grass	[31,33]
**62**	Gibellulic acid	C_15_H_20_O_3_	*M. chinensis* Maxim	Above ground part	[28]
**63**	3,4-Dihydroxy-β-ionone	C_13_H_20_O_3_	*M. chinensis* Maxim	Aboveground par	[7]
**64**	Perilloxin	C_16_H_18_O_4_	*M. chinensis* Maxim	Above ground part	[7]
**65**	Oleanolic acid	C_30_H_48_O_3_	*M. chinensis* Maxim *M. chinensis* ‘Jiangxiangru’	Above ground part, Whole grass	[28,30]
**66**	2α,3α,24-Trihydroxyolea-12en-28oic acid	C_30_H_48_O_5_	*M. chinensis* Maxim	Above ground part	[28]
**67**	2α,3β,24-Trihydroxyolea-12en-28oic acid	C_30_H_48_O_5_	*M. chinensis* Maxim	Above ground part	[28]
**68**	Ursolic acid	C_30_H_48_O_3_	*M. chinensis* Maxim *M. chinensis* ‘Jiangxiangru’	Whole grass	[26,34]
**69**	Betulinic acid	C_30_H_48_O_3_	*M. chinensis* ‘Jiangxiangru’	Whole grass	[34]
phenolic acids					
**70**	4-Hydroxybenzaldehyde	C_7_H_6_O_2_	*M. chinensis* Maxim	Above ground part	[7]
**71**	4-Hydroxy-3,5-dimethoxybenzaldehyde	C_9_H_10_O_4_	*M. chinensis* Maxim	Above ground part	[7]
**72**	*p*-Hydroxybenzoic acid	C_7_H_6_O_3_	*M. chinensis* ‘Jiangxiangru’	Whole grass	[30]
**73**	Paraben-β-D-Glucopyranoside	C_13_H_16_O_8_	*M. chinensis* ‘Jiangxiangru’	Whole grass	[32]
**74**	Syringic acid	C_9_H_10_O_5_	*M. chinensis* ‘Jiangxiangru’	Whole grass	[30]
**75**	4-Methoxyphenol	C_7_H_8_O_2_	*M. chinensis* Maxim	Stems and leaves	[31]
**76**	4-Hydroxy-2,6-dimethoxyphenyl-β-D-glucopyranoside	C_14_H_20_O_9_	*M. chinensis* ‘Jiangxiangru’	Whole grass	[32]
**77**	4-Hydroxy-3,5-dimethoxyphenyl-β-D-glucopyranoside	C_14_H_20_O_9_	*M. chinensis* ‘Jiangxiangru’	Whole grass	[32]
**78**	3,4,5-Trimethoxyphenyl-β-D-glucopyranoside	C_15_H_22_O_9_	*M. chinensis* ‘Jiangxiangru’	Whole grass	[32]
**79**	Isovanillyl alcohol-7-*O*-β-D-glucopyranoside	C_14_H_20_O_8_	*M. chinensis* Maxim	Stems and leaves	[31]
**80**	Vanillyl alcohol-7-*O*-β-D-glucopyranoside	C_14_H_20_O_8_	*M. chinensis* Maxim	Stems and leaves	[31]
**81**	Gastrodin	C_13_H_18_O_7_	*M. chinensis* Maxim	Stems and leaves	[31]
**82**	3-(*O*-β-D-glucopyranosyl)-α-(*O*-β-D-glucopyranosyl)-4-hydroxyphenylethanol	C_20_H_30_O_13_	*M. chinensis* Maxim	Stems and leaves	[31]
**83**	4-((*R*)-hydroxy((*R*)-2-(3-hydroxy-4-(hydroxymethyl)-phenyl)-propoxy) methyl)-2methylphenol	C_18_H_22_O_5_	*M. chinensis* Maxim	Stems and leaves	[31]
**84**	3-(3,4-dihydroxyphenyl) acrylic acid 1-(3,4-dihydroxyphenyl)-2-methoxycarbonylethyl ester	C_18_H_22_O_5_	*M. chinensis* Maxim	Above ground part	[9]
**85**	Methylrosmarinate	C_19_H_18_O_8_	*M. chinensis* Maxim	Stems and leaves	[31]
**86**	4′-Hydroxy-benzyl benzoate-4-*O*-β-D-glucopyranoside	C_20_H_22_O_9_	*M. chinensis* Maxim	Above ground part	[9]
**87**	3′-Hydroxy-4′-methoxy-benzyl benzoate-4-*O*-β-D-glucopyranoside	C_21_H_24_O_10_	*M. chinensis* Maxim	Above ground part	[9]
**88**	Amburoside A	C_20_H_22_O_10_	*M. chinensis* Maxim	Above ground part	[9]
**89**	4-[[(4-hydroxybenzoyl) oxy]-methyl]-phenyl-β-D-glucopyranoside	C_20_H_22_O_9_	*M. chinensis* Maxim	Stems and leaves	[31]
**90**	4-[[(2′,5′-dihydroxybenzoyl) oxy]-methyl]-phenyl-*O*-β-D-glucopyranoside	C_20_H_22_O_10_	*M. chinensis* Maxim	Above ground part	[9]
**91**	3′-Hydroxyphenyl-3,4,5-trimethylgallate	C_16_H_16_O_6_	*M. chinensis* Maxim	Above ground part	[28]
**92**	2,5-Dimethoxyphenethyl-3,4,5-trimethoxybenzoate	C_20_H_24_O_7_	*M. chinensis* Maxim	Stems and leaves	[31]
**93**	Mosla chinensis glycoside B1	C_21_H_28_O_9_	*M. chinensis* Maxim	Stems and leaves	[31]
**94**	Cucurbitoside D	C_25_H_30_O_13_	*M. chinensis* Maxim	Above ground part	[9]
**95**	Agrimonolide-6-*O*-β-D-glucopyranside	C_24_H_28_O_10_	*M. chinensis* Maxim	Above ground part	[28]
phenylpropanoids					
**96**	3-Hydroxyestragole-β-D-glucopyranoside	C_16_H_22_O_7_	*M. chinensis* ‘Jiangxiangru’	Whole grass	[32]
**97**	3-Hydroxy-4-methoxycinnamaladehyde	C_10_H_10_O_3_	*M. chinensis* Maxim	Above ground part	[7]
**98**	Methyl caffeate	C_10_H_10_O_4_	*M. chinensis* Maxim	Stems and leaves	[31]
**99**	Methyl-3-(3′,4′-dihydroxyphenyl) lactate	C_10_H_12_O_5_	*M. chinensis* ‘Jiangxiangru’	Whole grass	[33]
**100**	(*S*)-Pencedanol-7-*O*-β-D-glucopyranoside	C_20_H_26_O_10_	*M. chinensis* ‘Jiangxiangru’	Whole grass	[33]
**101**	(-)-5-Methoxyisolariciresinol	C_21_H_26_O_7_	*M. chinensis* ‘Jiangxiangru’	Above ground part	[29]
**102**	Lyoniresinol	C_22_H_28_O_8_	*M. chinensis* ‘Jiangxiangru’	Above ground part	[29]
**103**	Pinoresinol	C_20_H_22_O_6_	*M. chinensis* ‘Jiangxiangru’	Above ground part	[29]
**104**	Isoeucommin A	C_27_H_34_O_12_	*M. chinensis* ‘Jiangxiangru’	Above ground part	[29]
**105**	Syringaresinol-4′-*O*-β-D-monoglucoside	C_28_H_36_O_3_	*M. chinensis* Maxim	Stems and leaves	[31]
**106**	Episyringaresinol-4-*O*-β-D-glucopyranoside	C_28_H_36_O_13_	*M. chinensis* ‘Jiangxiangru’	Above ground part	[29]
**107**	(7*R*,8*S*)-dihydrodehydrodiconiferyl alcohol 9-*O*-β-D-glucopyranoside	C_26_H_34_O_11_	*M. chinensis* Maxim	Stems and leaves	[31]
**108**	Methyl salvianolate C	C_27_H_22_O_10_	*M. chinensis* Maxim	Above ground part	[24]
**109**	Rel-(7*R*,8*S*)-3,3′,5-trimethoxy-4′,7-epoxy-8,5′-neolignan-4,9,9′-triol 9-*O*-β-D-glucopyranoside	C_27_H_36_O_12_	*M. chinensis* Maxim	Stems and leaves	[31]
**110**	Monomethyl lithospermate	C_28_H_24_O_12_	*M. chinensis* Maxim	Above ground part	[9]
**111**	Dimethyl lithospermate	C_29_H_26_O_12_	*M. chinensis* Maxim	Above ground part	[24]
**112**	Sebestenoids C	C_36_H_30_O_14_	*M. chinensis* Maxim	Above ground part	[24]
**113**	Hyprhombin B methyl ester	C_27_H_22_O_10_	*M. chinensis* Maxim	Above ground part	[9]
**114**	Dimethyl clinopodic acid C	C_29_H_26_O_12_	*M. chinensis* Maxim	Above ground part	[24]
others					
**115**	Linoleic acid	C_18_H_32_O_2_	*M. chinensis* Maxim	Above ground part	[28]
**116**	Oleic acid	C_18_H_34_O_2_	*M. chinensis* Maxim	Above ground part	[28]
**117**	6-Methyltritriacontane	C_34_H_70_	*M. chinensis* Maxim	Whole grass	[26]
**118**	Prunasin	C_14_H_17_NO_6_	*M. chinensis* ‘Jiangxiangru’	Whole grass	[33]
**119**	Sambunigrin	C_14_H_17_NO_6_	*M. chinensis* ‘Jiangxiangru’	Whole grass	[33]
**120**	Benzyl-D-glucopyranoside	C_13_H_18_O_6_	*M. chinensis* ‘Jiangxiangru’	Whole grass	[33]
**121**	Adenosine	C_10_H_13_N_5_O_4_	*M. chinensis* ‘Jiangxiangru’	Whole grass	[32]
**122**	Indole-3-carboxylic acid β-D-glucopyranosyl	C_15_H_17_NO_7_	*M. chinensis* Maxim *M. chinensis* ‘Jiangxiangru’	Stems and leaves, Whole grass	[30,31]
**123**	β-Sitosterol	C_29_H_50_O	*M. chinensis* Maxim *M. chinensis* ‘Jiangxiangru’	Whole grass	[26,30]

**Table 4:** Summary of pharmacological activities of MH extracts/compound.

Pharmacological Activities	Compounds/Extracts	Model/Method	Result/Mechanism	Dosage	Ref.
Anti-bacterial	Volatile oils	Ten bacteria (*Staphylococcus aureus*, *Staphylococcus epidermidis*, *Shigella dysenteriae*, *F’s dysentery bacillus*, *Shigella sonnei*, *Salmonella typhi*, *Salmonella paratyphi* B, *Salmonella typhimurium*, *Escherichia coli*, and *Proteus vulgaris*)	No bacterial growth	78–312 mg/L	[52]
Antiviral	Volatile oils	CEF (NDV-induced)	↓Number of cell lesions	0.3, 0.7 g/L	[53]
	Volatile oils	Vero cells; mice (A3 virus-induced)	↓Blood coagulation titer and virus amplification; treat pneumonia in mice	4.9 mg/L; 100 μg/g/d	[54]
	Flavonoids	ICR male mice (H1N1 virus-infected)	↓Lung indices, IL-2, SOD, GSH, TLR3, TLR7, MyD88, TRAF3, and NF-κB p65; ↑IL-6, TNF-α, IFN-γ, and NO	144, 288, 576 mg/kg	[39]
	Compound **84**	H1N1 influenza virus	89.2% inhibition rate	100 μmol/L	[9]
	Compound **110**	H1N1 influenza virus	98.6% inhibition rate	100 μmol/L	[9]
Anti-inflammatory	Water extract	ICR mice with allergic inflammation (mast cell-mediated); SD rats peritoneal mast cells (activated by compound 48/80 or IgE)	↓Intracellular calcium levels, histamine, TNF-α, IL-6, and IL-8	10–1000 mg/kg; 0.01–10 mg/ml	[55]
	Methanol extract	Male c57BL/6 mice (DSS-induced)	↓NO, PGE2, TNF-α, IL-6, IL-1β, ROS, and MDA; ↑Activities of CAT, SOD, and T-AOC	27.9, 111.6 mg/kg/day	[56]
	Volatile oils	ICR mice with intestinal inflammation (LPS-induced)	↓TNF-α, IL-1β, IL-6, TLR4, NF-κB p65, and JNK; ↑IL-10, IFN-γ, and mRNA	0.06, 0.2, 0.5 mL/kg	[57]
Antioxidant	MP-1	Male BALB/c mice	Over 80% free radical scavenging rate	0.5–20 mg/mL	[50]
Analgesic	Volatile oils	KM male mice	↑Pain threshold in mice	0.1–0.3 mg/kg	[58]
Sedative	Volatile oils	KM mice	↑Hypnotic effect of pentobarbital sodium	0.1, 0.3 mL/kg	[59]
Antipyretic	Volatile oils; water extract	SD rats (LPS-induced)	↓Anal temperature; ↓PGE2, TNF-α, cAMP, IL-1β, and MPO	1.0, 6.2 g/kg	[12]
Regulate gastrointestinal motility	Volatile oils	SD rats	↑Body weight, gastric emptying rate, intestinal propulsion rate, and serum gastrin content ↓Fecal wet weight, intestinal water content, and serum motilin	0.1, 0.3, 0.6 g/mL	[60]
	Volatile oils	duodenum	Double regulation	0.03, 0.06‰	[61]
Immunomodulatory	Volatile oils	KM mice	↑Carbon clearance index, thymus, and spleen weight	43, 130, 390 mg/kg	[13]
	MP	KM mice (CTX-induced)	↓MDA; ↑Thymus and spleen indices	200, 400 mg/kg	[62]
Insecticidal	Water extract	TV	Insect body rupture	62.5 mg/mL	[63]
	Volatile oils	*A. gossypii*	More than 85% mortality rate	5 μL	[14]
	Volatile oils	*A. albopictus* (larvae and pupae)	Poisoning larvae, repelling adult mosquitoes	LC_50_ = 78.8 μg/mL LC_50_ = 122.6 μg/mL	[64]
Inhibit α-glucosidase activity	75% Ethanol extraction of ethyl acetate extract; volatile oils	α-Glucosidase activity inhibition test	Inhibition rate of 84.2%, nearly 100%	1.0 mg/mL; 0.5 mg/mL	[65]

Arrow ↓ means decrease, Arrow ↑ means increase.

## Data Availability

No new data were created or analyzed in this study. Data sharing is not applicable to this article.

## Abbreviations

TCM	traditional Chinese medicine
matK	megakaryocyte-associated tyrosine kinase
ITS2	internal transcribed spacer 2 database
DPPH	1,1-diphenyl-2-picrylhydrazyl radical
ABTS	2,2′-azino-bis (3-ethylbenzothiazoline-6-sulfonic acid)
FRAP	ferric reducing antioxidant power
CMP	crude polysaccharide CMP
MP	polysaccharide MP
MP-1	polysaccharide MP-1
MP-A40	polysaccharide MP-A40
IL	interleukin
TNF-α	tumor necrosis factor-α
SOD	superoxide dismutase
GSH	glutathione
IgE	immunoglobulin E
ROS	reactive oxygen species
ConA	concanavalin A
NDV	Newcastle disease virus
CEF	chicken embryo fibroblast cells
T-AOC	total antioxidant capacity
CAT	catalase
GSH-PX	glutathione peroxidase
MDA	malondialdehyde
LPS	lipopolysaccharide
TLR	toll-like receptor
MyD88	myeloiddifferentiationfactor88
TRAF3	TNF receptor-associated factor 3
NF-κB	nuclear factor kappa-B
IFN-γ	interferon-γ
PGE2	prostaglandin E2
NO	nitric oxide
JNK	c-Jun N-terminal kinase
mRNA	messenger RNA
cAMP	adenosine cyclophosphate
MPO	myeloperoxidase
DSS	dextran sulfate sodium salt
CTX	cyclophosphamide
TV	trichomonas vaginalis
Nrf2/HO-1	nuclear factor erythroid2-related factor 2/heme oxygenase-1
LC_50_	half lethal concentrations

## References

[1] Ye M.Q., Wu M.H., Ma Z.G., Cao H., Zhang Y. (2023). HPLC fingerprint and multi-components quantitative analysis of Moslae Herba. Chin. J. Pharm. Anal..

[2] Yao Y., Xu J., Huang G.X., Zhang T.J., Liu C.X. (2020). Research progress of Moslae Herba and predictive analysis on its Q-marker. Chin. Tradit. Herb. Drugs.

[3] Shan F., Zhang W.X., Zhang S.H., Liu H., Zhan Z.L. (2023). Herbal textual research on Moslae Herba in famous classical formulas. Chin. J. Exp. Tradit. Med. Formulae.

[4] China Pharmacopoeia Committee (2005). Pharmacopoeia of the People’s Republic of China.

[5] Mao Y., Li Z.G., Cao J.L. (2008). Analysis of volatile oil composition of *Mosla chinensis*. J. Zhejiang A&F Univ..

[6] Yu H., Hu J., Bai F.P., Qin K.M. (2017). Analysis of volatiles from bark of Moslae Herba by gas chromatography-mass spectroscopy. Guid. J. Tradit. Chin. Med. Pharm..

[7] Xu J.Y., Liu R., Huang X.J., Liao M.C., Li J. (2022). Chemical constituents of *Mosla chinensis*. J. Chin. Med. Mater..

[8] Hu H.W., Xie X.M., Zhang P.Z., Shun R.G. (2010). Study on the flavonoids from *Mosla chinensis* ‘Jiangxiangru’. J. Chin. Med. Mater..

[9] Du J.C., Yang L.Y., Shao L., Yu F., Li R.T., Zhong J.D. (2021). Study on phenolic acid compounds from acrial parts of *Mosla chinensis* and its anti-influenza activity. J. Chin. Med. Mater..

[10] Peng L., Xiong Y., Wang M., Han M., Cai W., Li Z. (2018). Chemical composition of essential oil in *Mosla chinensis* Maxim cv. ‘Jiangxiangru’ and its inhibitory effect on *Staphylococcus aureus* biofilm formation. Open Life Sci..

[11] Luo Q., Deng Z.Y., Hong T., Guo S.Y., Chen Y.R., Luo L.M., Ma Q.T., Xu L., Liu Z.Y. (2022). Chemical composition analysis of volatile oils and fatty acids from *Mosla chinensis* seeds and antioxidant activity. Jiangxi Sci..

[12] Sun D.Y., Gao H., Wang X.T., Yan L., Pang B. (2018). Antipyretic and anti-inflammatory effects of *Mosla chinensis* based on integration processing technology of origin. Chin. Tradit. Herb. Drugs.

[13] Ge B. (2005). Studies on Extraction and Pharmacodynamics of Volatile Oil in *Mosla chinensis* Maxim. Ph.D. Thesis.

[14] Gao Y. (2013). Studies on biological activity of volatile oil of *Mosla chinensis* Maxim on cotton aphid. Agric. Sci. Technol. Inf..

[15] Liu H.N., Jiang X.X., Naeem A., Chen F.C., Wang L., Liu Y.X., Li Z., Ming L.S. (2022). Fabrication and characterization of β-cyclodextrin/*Mosla chinensis* essential oil inclusion complexes: Experimental design and molecular modeling. Molecules.

[16] Zheng M.D., Zhang C., Ma R.L., Zhang Y., Wang X.J., Qin B. (2022). Identification of Moslae Herba and its adulterants based on matk, ITS2 and its secondary structure. Chin. J. Mod. Appl. Pharm..

[17] Lu Y., Liu Y., Liu X.Y., Feng Z.K. (2022). Development status of genuine characteristic medicinal materials in Jiangxi province. J. Jiangxi Univ. Chin. Med..

[18] Zhang S.W., Liu X.W., Hu S.F., Cao L., Peng L.L. (2004). The characteristics of growing and developing and the cultivation technology of *Mosla chinensis* Maxim. Acta Agric. Univ. Jiangxiensis.

[19] Huang L.Q., Guo L.P., Zhan Z.L. (2020). Standard Compilation of Genuine Medicinal Materials.

[20] Zhang M.J., Wang W.L., Wang P. (2020). Comparative study on clinical application of Xiangru (Molae Herba) in ancient and modern times. Shandong J. Tradit. Chin. Med..

[21] Yuan H., Sun Y.X. (2018). Treating 200 cases of cold of the Shushi type with Xinjia Xiangru Yin. Clin. J. Chin. Med..

[22] Zhang S. (2016). Treating 86 cases of acute pharyngeal conjunctival fever in children with Xinjia Xiangru Yin. Inn. Mong. J. Tradit. Chin. Med..

[23] Yao J.C., Ying Y.F., Zheng H.W., Hu G.H., Lin Y.P. (2005). Study of Chaihu-Xiangru decoction on acute upper respiratory infection in summer. Mod. J. Integr. Tradit. Chin. West. Med..

[24] Yang L.Y. (2021). Studies on the Chemical Constituents and Anti-Influenza Activity of *Elsholtzia bodinieri* and *Mosla chinensis*. Ph.D. Thesis.

[25] Sun L.P., Zheng S.Z., Fu Z.S., Shen X.W. (1995). Flavonoids from *Mosla chinensis* Maxim. J. Northwest Norm. Univ. Nat. Sci..

[26] Zheng S.Z., Sun L.P., Shen X.W. (1996). Chemical constituents of *Mosla chinensis* Maxim. J. Integr. Plant Biol..

[27] Yang C.X., Kang S.H., Jing L.T., Zheng S.Z. (2003). Flavonoids from *Mosla chinensis* Maxim. J. Northwest Minor. Univ. Nat. Sci..

[28] Du J.C. (2022). Studies on the Chemical Constituents and Anti-Influenza Activities of *Elsholtzia densa* and *Mosla chinensis*. Ph.D. Thesis.

[29] Zou Y., Pan J.P., Zhou M. (2021). Analysis of ethyl acetate constituents and inhibit α-glucosidase activity of *Mosla chinensis* ‘Jiangxiangru’. China Food Saf. Mag..

[30] Liu H., Zhang D., Luo Y.M. (2010). Studies on Chemical constituents of *Mosla chinensis* ‘Jiangxiangru’. Chin. J. Exp. Tradit. Med. Formulae.

[31] Feng S.Y. (2022). Studies on the Chemical Constituents and Anti-Influenza Activities of *Mosla chinensis* and *Prunella vulgaris*. Ph.D. Thesis.

[32] Shen J.J., Zhang D.M., Liu H., Luo Y.M. (2011). Polar constituents of *Mosla chinensis*. China J. Chin. Mater. Med..

[33] Liu H., Shen J.J., Zhang D.M., Luo Y.M. (2010). Studies on polar constituents of *Mosla chinensis* ‘Jiangxiangru’. Chin. J. Exp. Tradit. Med. Formulae.

[34] Shu R.G., Hu H.W., Zhang P.Z., Ge F. (2012). Triterpenes and flavonoids from *Mosla chinensis*. Chem. Nat. Compd..

[35] Lin W.Q., Liu J.Q., Lan R.F. (1999). Studies on the chemical constituents of essential oil and its antibacterial effect of *Mosla chinensis* Maxim Growing in Fujian Province. J. Fujian Teach. Univ. Nat. Sci. Ed..

[36] Lin C.L., Cai J.Z., Lin G.Y. (2012). Chemical constituents study of volatile oils from the *Mosla chinensis* Maxim in Zhejiang province. Chin. Arch. Tradit. Chin. Med..

[37] Zheng S.Z., Zheng M.Y., Dai R., Yang C.X., Shen T. (2001). Studies on the chemical component of essential oil of *Mosla chinensis* maxin by means of supercritical fluid extraction. J. Northwest Norm. Univ. Nat. Sci..

[38] Chen Y.M. (2016). Based on Chromatography-Mass Spectrometry Technology Research Chemical Constituents of *Mosla chinensis* Maxim cv. ‘Jiangxiangru’. Ph.D. Thesis.

[39] Zhang X.X., Wu Q.F., Yan Y.L., Zhang F.L. (2018). Inhibitory effects and related molecular mechanisms of total flavonoids in *Mosla chinensis* Maxim against H1N1 influenza virus. Inflamm. Res..

[40] Hu Z.X., Tong L., Geng Y.M., Yang Q., Hou J.F. (2022). A review on pharmacological activities and preparations of luteolin. Clin. J. Chin. Med..

[41] Jiang Y.L., Li W.Y., Feng S., Liu Jin H., Zhai G.Y. (2023). Research progress on structural modification and biological activity of luteolin. Chin. Tradit. Herb. Drugs.

[42] Feng Y.L., Li Ke Liu J.H., Zhai G.Y. (2020). Study on synthesis and biological activities of quercetin-3-*O*-amide derivatives. Chin. Tradit. Herb. Drugs.

[43] Marinelli L., Fornasari E., Eusepi P., Ciulla M., Genovese S., Epifano F., Fiorito S., Turkez H., Örtücü S., Mingoia M. (2019). Carvacrol prodrugs as novel antimicrobial agents. Eur. J. Med. Chem..

[44] Srinivasulu C., Ramgopal M., Ramanjaneyulu G., Anuradha C.M., Suresh Kumar C. (2018). Syringic acid (SA)-a review of its occurrence, biosynthesis, pharmacological and industrial importance. Biomed. Pharmacother..

[45] Xiao G., Tang R., Yang N., Chen Y. (2023). Review on pharmacological effects of gastrodin. Arch. Pharm. Res..

[46] Huang Q., Ouyang D.S., Liu Q. (2021). Isoeucommin A attenuates kidney injury in diabetic nephropathy through the Nrf2/HO-1 pathway. FEBS Open Bio.

[47] Guo X.H., Pang L., Gao C.Y., Meng F.L., Jin W. (2023). Lyoniresinol attenuates cerebral ischemic stroke injury in MCAO rat based on oxidative stress suppression via regulation of Akt/GSK-3β/Nrf2 signaling. Biomed. Pharmacother..

[48] Cao X.Y., Liu X.F., Yin Y.X., Wang B., Huang W.H. (2021). The solubility characteristics and antidepressant activity of β-sitosterol and its derivatives. Ningxia Med. J..

[49] Li J.E. (2014). Structure Analysis and Functional Properties of Polysaccharides from *Mosla chinensis* Maxim cv. ‘Jiangxiangru’ and Two Arabic Gums. Ph.D. Thesis.

[50] Li J.E., Nie S.P., Xie M.Y., Li C. (2014). Isolation and partial characterization of a neutral polysaccharide from *Mosla chinensis* Maxim cv. ‘Jiangxiangru’ and its antioxidant and immunomodulatory activities. J. Funct. Foods.

[51] Yi Y., Liu H., Chen Z.W., Luo Y.M., Wan C.Y. (2012). Determination of mineral elements in different part of *Mosla chinensis* with ICP-MS. Chin. J. Exp. Tradit. Med. Formulae.

[52] Feng Y., Liu J. (2009). Effects of volatile oil from *Mosla chinensis* Maxim on bacteriostasis and immune response. Biotic Resour..

[53] Ge B., Lu X.Y., Jiang H.M., Fang J., Mo J., Xie F.J. (2005). Antiviral effect of volatile oil from *Mosla chinensis* Maxim on NDV in vitro. J. Tradit. Chin. Vet. Med..

[54] Yan Y.F., Chen X., Yang X.Q., Dong C., Wu S.Y., Chen J.B., Wang C.Y. (2002). Inhibition of *Mosla chinensis* volatile oil (Mcvo) on influenza virus A3. J. Microbiol..

[55] Kim H.H., Yoo J.S., Shin T.Y., Kim S.H. (2012). Aqueous extract of *Mosla chinensis* inhibits mast cell-mediated allergic inflammation. Am. J. Chin. Med..

[56] Wang X., Cheng K., Liu Z., Sun Y., Zhou L., Xu M., Dai X., Xiong Y., Zhang H. (2021). Bioactive constituents of *Mosla chinensis* cv. ‘Jiangxiangru’ ameliorate inflammation through MAPK signaling pathways and modify intestinal microbiota in DSS-induced colitis mice. Phytomedicine.

[57] Yang H.C. (2021). Effect and Molecular Mechanism of Essential Oil of *Mosla chinensis* Maxim on Improving Diarrhea in Mice. Ph.D. Thesis.

[58] Gong M.X. (2000). Comparison of pharmacological action of volatile oil between green *Mosla chinensis* Maxim and *Mosla chinensis* ‘Jiangxiangru’. Beijing J. Tradit. Chin. Med..

[59] Wu T.K., Zhou Y.L., Zhou S.Q., Wang X.D. (1992). Comparative study on the pharmacological effects of volatile oils of *Mosla chinensis* Maxim, *Mosla chinensis* ‘Jiangxiangru’, *Origanum vulgare* Linn and *Elsholtzia splendens* Nakai. J. Chin. Med. Mater..

[60] Sun D.Y., Gao H. (2017). Effect of Moslae Herba volatile oil on rat models with the spleen and the stomach stranded by dampness. Chin. Tradit. Pat. Med..

[61] Jiang H.M. (2007). Studies on the Volatile Oil, Bioactivities and Germplasm Innovation Technique of *Mosla chinensis* Maxim in Hunan. Ph.D. Thesis.

[62] Li J.E., Nie S.P., Xie M.Y., Huang D.F., Wang Y.T., Li C. (2013). Chemical composition and antioxidant activities in immumosuppressed mice of polysaccharides isolated from *Mosla chinensis* Maxim cv. ‘Jiangxiangru’. Int. Immunopharmacol..

[63] Zheng L.L., Cui y Qin Y.H., Ren Y.X., Liu X., Tao L., Dai X.D. (2009). Effect of *Mosla chinensis* Maxim on *Trichomonas vaginalis* in vitro. J. Dalian Med. Univ..

[64] Chen F.F., Peng Y.H., Zeng D.Q., Zhang Y., Qin Q.H., Huang Y. (2010). Composition and bioactivity of the essential oils from *Mosla chinensis* against *Aedes albopictus*. Chin. J. Vector Biol. Control.

[65] Wang M., Li Z.M., Peng L. (2015). α-Glucosidase inhibition of *Mosla chinensis* Maxim cv. ‘Jiangxiangru’ extracts. J. Jiangxi Sci. Technol. Norm. Univ..

[66] Li Z.M., Sun Y.M., Wang M., Peng L., Ren P. (2014). Study on bacteriostatic activity of *Mosla chinensis* ‘Jiangxiangru’ extracts. Sci. Technol. Food Ind..

[67] Qi W.W., Shen Y.T., Chen C.Y., Chen J.Y., Wan C.P. (2018). Antifungal mechanisms of the active ingredients carvacrol of *Mosla chinensis* ‘Jiangxiangru’ against *Penicillium digitatum*. Mod. Food Sci. Technol..

[68] Cao L., Si J.Y., Liu Y., Sun H., Jin W., Li Z., Zhao X.H., Pan R.L. (2009). Essential oil composition, antimicrobial and antioxidant properties of *Mosla chinensis* Maxim. Food Chem..

[69] Zhang Q., Wu Q.F., Zhu W.R., Liu X. (2014). Anti-oxidation effects of total flavonoids from *Mosla chinensis* Maxim. Chin. Arch. Tradit. Chin. Med..

[70] Sun T.T. (2016). Extraction Purification and Antioxidant Activity of Polysaccharides from *Mosla chinensis* Maxim cv. ‘Jiangxiangru’. Ph.D. Thesis.

[71] Li J.E., Cui S.W., Nie S.P., Xie M.Y. (2014). Structure and biological activities of a pectic polysaccharide from *Mosla chinensis* Maxim cv. ‘Jiangxiangru’. Carbohydr. Polym..

[72] Zhang Y.X. (2019). Study on the Processing Mechanism Moslae Herba Produced by Ginger Juice. Ph.D. Thesis.

[73] Ma Q.T., Xu L., Zhu Y.C., Xu M.T., Zhang W.K., Liu Z.Y. (2024). Effects of polysaccharide content and anti-inflammatory, hemostatic, and antioxidant activities before and after preparation of *Mosla chinensis*-Jiangxiangru. Chin. J. Comp. Med..

[74] Chen Z.D., Lu X.Y., Jiang H.M., Zhou H., F J., Peng T., Liu A.P. (2007). Study on the effect of *Mosla chinensis* Maxim volatile oil and several common food preservatives on chilled meat preservation. Meat Res..

[75] Sun L.F. (1993). Preparation and application in tobacco flavor of *Mosla chinensis* Maxim extractum. Flavour. Fragrance Cosmet..

[76] Wang X.D., Sun H.B., Wang X.Z., Huo L.P., Chen W.H., Kong L.J. (1995). Observation of natural air fresheners and their effects. Chin. J. Public Health.

